# Work-Life Balance, Job Satisfaction, and Job Performance of SMEs Employees: The Moderating Role of Family-Supportive Supervisor Behaviors

**DOI:** 10.3389/fpsyg.2022.906876

**Published:** 2022-06-21

**Authors:** Perengki Susanto, Mohammad Enamul Hoque, Taslima Jannat, Bamy Emely, Mega Asri Zona, Md Asadul Islam

**Affiliations:** ^1^Department of Management, Universitas Negeri Padang, Padang, Indonesia; ^2^BRAC Business School, BRAC University, Dhaka, Bangladesh; ^3^Faculty of Economics and Management, National University of Malaysia, Bangi, Malaysia

**Keywords:** work-life balance, job satisfaction, job performance, family-supportive supervisor behaviors, Indonesia

## Abstract

Even though studies on work-life balance and family-supportive supervisor behaviors are prevalent, there are few studies in the SME setting, and the implications are yet unexplained. Thus, the study examines the effect of work-life balance on the performance of employees in SMEs, along with the mediating role of job satisfaction and the moderating role of family-supportive supervisor behaviors. We have developed a conceptually mediated-moderated model for the nexus of work-life balance and job performance. We collected data from SMEs and employed SEM-PLS to test the research hypothesis and model. Empirical results demonstrate that work-life balance positively influences job satisfaction and performance. Our empirical findings also revealed that job satisfaction partially mediates the relationship between work-life balance and job performance. We also found that when FSSB interacts with work-life balance and job satisfaction, it moderates the relationship between work-life balance and job performance and job satisfaction and job performance. Hence, our findings provide exciting and valuable insights for research and practice.

## Introduction

The importance of Small and Medium Enterprises (SMEs) in the global and national economies is worth mentioning, considering their role in creating employment and contributing to GDP. According to a World Bank (2020) survey on SMEs, the sector accounts for 90% of businesses and 50% of jobs globally. According to the report, this sector contributes more than 40% of GDP and creates 70% employment in developing economies. The SME sector is rapidly expanding in Indonesia, and around 63 million SMEs operate (Surya et al., 2021). Of those, 62 million are classified as medium-sized firms, and 0.75 million are classified as small businesses. SMEs are divided into four categories: household businesses with 1–5 workers; small and medium businesses with 6–19 workers; medium-sized companies with 20–29 workers; and large companies with more than 100 workers (Badan Pusat Statistik, 2020). More importantly, the sector contributes 61.07% of the country’s total GDP and provides 97% of the entire employment (ILO, 2019; Kementerian Koperasi dan UKM Republik Indonesia, 2019; Pramono et al., 2021).

Given the importance of SMEs in the economy, it is necessary to maintain and sustain the sector’s human resource performance. A strand of the literature highlighted that firm-specific factors and the environment impact employee performance. Another strand of the literature highlighted that the performance of an employee could be influenced by cognitive factors, such as individual quality (Luthans et al., 2007), supervisor support, work-life balance (Talukder et al., 2018), cognitive abilities, personality (Kanfer and Kantrowitz, 2005), leadership, and family supportive supervisor behaviors (Walumbwa et al., 2010; Wang et al., 2013; Kim et al., 2015). Although all these factors are important determinants, the current study argues that work-life balance and family supportive supervisor behavior are more important than employees’ involvement in every possible business activity of SMEs.

In the SME world, the working hours are different from those in larger firms. SMEs demand longer hours from employees. Therefore, it is difficult for employees to balance work and personal life. Some of the time, they also failed to maintain social and personal life due to high engagement and stress at work. The entanglements between work and family are a significant source of psychological discomfort for employees (Cegarra-Leiva et al., 2012; Lamane-Harim et al., 2021). This could lead to job dissatisfaction and poor job performance. Hence, the employee turnover and the intention to quit. On the other hand, Haar et al. (2014) stated that WLB has a positive impact on one’s achievements, including performances. Similarly, increased job satisfaction impacts performance (Luthans et al., 2007; Walumbwa et al., 2010). Positive job satisfaction will increase employee capacity, which, if appropriately managed, will have a good impact on the employee’s job performance (Luthans et al., 2007).

However, in the competitive market, being a small team, the SMEs may not be able to afford to lose their skilled and knowledgeable employees as they are involved in product innovation and product sales. In order to facilitate work-life balance, SMEs indeed need to deploy the WLB’s supportive culture. Lamane-Harim et al. (2021) suggest that practices or the introduction of WLBSC could influence job satisfaction and organizational commitment. These factors ultimately determine employee performance in SMEs and their sustainability (e.g., Cuéllar-Molina et al., 2018). In the practices of WLBSC, family-supportive supervisor behaviors could play an important role, as family-supportive supervisor behaviors are expected to influence outcomes related to one’s performance (Wang et al., 2013). In previous studies, supportive family supervisor behaviors were associated with job satisfaction and job performance (Greenhaus et al., 2012; Wang et al., 2013; Heras et al., 2021). Past studies also suggest the mediating role of work-life balance supportive culture in SMEs. However, since the work-life balance supportive culture is a contextual factor and a new introduction into the working environment, it is expected to increase or decrease the extent of the relationship between work-life balance (WLB) and job satisfaction and the relationship between work-life balance (WLB) and job performance. It also raises the question of how moderation affects the existing relationship between work-life balance (WLB) and job satisfaction and the relationship between work-life balance (WLB) and job performance. However, past studies have not investigated the moderating role of family-supportive supervisor behaviors (e.g., Greenhaus et al., 2012; Wang et al., 2013; Heras et al., 2021; Lamane-Harim et al., 2021).

Past studies on work-life balance have primarily focused on large firms. Several other studies have recommended more studies of this topic in SMEs (Lavoie, 2004; Cegarra-Leiva et al., 2012). Recently, Lamane-Harim et al. (2021) have researched work-life balance and WLBSC on Spanish SMEs. Furthermore, most research analyzing the relationships between WLBSC and employee outcome has been conducted in the United States. Moreover, national culture can also affect the intensity of the link between WLB practices and their effects on employee outcomes (Spector et al., 2007; Poelmans et al., 2005; Cegarra-Leiva et al., 2012; Lucia-Casademunt et al., 2015; Ollier-Malaterre and Foucreault, 2017; Putnik et al., 2020; Kelley et al., 2021). Thus, the current study fills the research gap by examining the moderating role of family-supportive supervisor behaviors on the relationship between work-life balance (WLB) and job satisfaction and the relationship between work-life balance (WLB) and job performance. To fulfill these objectives, a review of the literature is carried out. The research hypotheses are developed, which are examined in an empirical study with a sample of employees of Indonesian SMEs in an industrial sector. The implications arising from the investigation are given in the final part. Henceforth, the current study will be beneficial to the SME sector in Indonesia alongside the literature.

## Literature Review

### Social Exchange Theory

According to the Social Exchange Theory (SET) (Blau, 1964), social exchange relationships rest on the norm of reciprocity (Gouldner, 1960). The theory argues that when one party provides a benefit to another, the recipient tends to reciprocate the favor by offering benefits and favorable treatment to the first party (Coyle-Shapiro and Shore, 2007). In an organizational behavior context, the social exchange theory is frequently used to explain the formation and maintenance of interpersonal relationships between employees and employers regarding reciprocation procedures (Chen et al., 2005; Rawshdeh et al., 2019). The theory explains why employees choose to be less or more engaged in their jobs (Lee and Veasna, 2013) and how the organizational support system influences subordinates’ creativity (Amabile et al., 2004) and other positive behavior.

**FIGURE 1:**
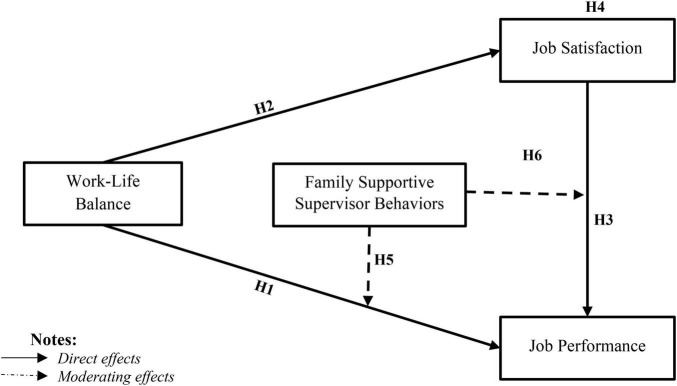
Conceptual research model.

Past studies have argued that when management provides benefits to employees, employees tend to feel indebted to the organization and make more substantial efforts to ensure its well-being and achieve its goal (Eisenberger et al., 2001; Vayre, 2019). Several studies found evidence in the work-life balance literature that when organizations or supervisors care about their employees’ personal and professional well-being, employees tend to reciprocate by helping them achieve their goals through improved performance (Campo et al., 2021). Therefore, based on the social exchange theory, this study argues that when organizations take care of the balance between employees’ personal and professional lives, employees’ perceived positive feelings increase their job satisfaction, and they are more inclined to reciprocate the favor through high job performance (Talukder et al., 2018). In such circumstances, the supervisor’s formal and informal support further increases employees’ perceived positive feelings toward the job and strengthens the relationship between work-life balance, job satisfaction, and job performance. We present a conceptual model in Figure 1, which illustrates the expected causal relationship among study variables.

### Job Performance

Employee job performance refers to an employee’s expertise in carrying out their duties in a way that helps the organization achieve its goals (Luthans et al., 2007, 2008; Nohe et al., 2014; Moonsri, 2018). It is also defined as an individual’s productivity compared to their coworkers on a variety of job-related behaviors and results (Babin and Boles, 1998; Aeknarajindawat and Jermsittiparsert, 2020). Performance is determined by the quality and quantity of work completed as part of an employee’s assigned responsibilities. Employee performance directly influences an organization’s financial and non-financial outcomes (Anitha, 2014). Thus, organizations need high-performing employees to achieve their corporate goals, vision, and mission and gain a competitive advantage (Thevanes and Mangaleswaran, 2018).

A business must have a persistent competitive advantage in the SME context with many competitors to compete with other companies in the same industry. While job stress has been shown to have a significant negative impact on employee performance, work overload, lack of work-life balance, management style, and job insecurity are some of the factors that contribute to increased job stress (Naqvi et al., 2013). Since SMEs need employees to work longer hours, it is possible that SMEs’ employees lack a healthy balance between work and family life, thereby impacting their job performance. Organizations are increasingly focusing on implementing a variety of HR practices and strategies, including work-life balance, on increasing employee job performance, as work-life balance is seen as one of the most important factors influencing job performance (Thevanes and Mangaleswaran, 2018). Previous research found ample evidence that work-life balance is essential to increasing employee job performance (Preena, 2021). Therefore, the role of work-life in influencing SME employees’ job performance should be determined to ensure the industry’s survival.

### Work-Life Balance, Job Satisfaction, and Job Performance

Work-life balance refers to balancing one’s professional work, family responsibilities, and other personal activities (Keelan, 2015; Kerdpitak and Jermsittiparsert, 2020). It refers to an employee’s sense of a balance between work and personal life (Haar et al., 2014). It represents how people fulfill or should fulfill their business and personal obligations so that an overlapping situation is avoided (Konrad and Mangel, 2000). The changing work patterns and the pressing demand for domestic chores have had an adverse impact on people’s work, social, and family lives (Barling and Macewen, 1992). Therefore, researchers suggested that the human resource management of an organization should develop effective policies such as adequate mentoring, support, flexible working hours, reducing workload, and many others that can reduce employees’ work-life conflict (Cegarra-Leiva et al., 2012) and positively influence their satisfaction (Allen et al., 2020) and performance (Hughes and Bozionelos, 2007).

Work-life balance is one of the most important issues that human resource management should address in organizations (Abdirahman et al., 2020). Regardless of their size, organizations should ensure that employees have adequate time to fulfill their family and work commitments (Abdirahman et al., 2020). A flexible working environment allows employees to balance personal and professional responsibilities (Redmond et al., 2006). Organizations that ignore the issue of work-life balance suffer from reduced productivity and employee performance (Naithani, 2010). Indeed, employees with a healthy work-life balance are generally grateful to their employers (Roberts, 2008). As a result, they put forth their best effort for the company as a gesture of gratitude, resulting in improved job performance (Ryan and Kossek, 2008). Thus, a high work-life balance employee could be highly productive and an excellent performer (French et al., 2020). Thus, based on these discussions and research findings, we developed the following hypothesis:

Hypothesis 1: Work-life balance has a positive effect on job performance.

Previous researchers have argued that satisfaction and success in family life can lead to success and satisfaction at work Victoria et al. (2019). Employees who are pleased with their personal and professional achievements are more likely to achieve the organizational goal (Dousin et al., 2019). While the work-life conflict has been shown to have a negative impact on employee job performance and satisfaction (Dousin et al., 2019), work-life balance has been found to improve employee satisfaction and job performance in various industries and countries (Mendis and Weerakkody, 2017; Thevanes and Mangaleswaran, 2018; Victoria et al., 2019; Obrenovic et al., 2020; Rini et al., 2020; Preena, 2021). It is documented that medical doctors’ job satisfaction and performance are influenced by their perceptions of flexible working hours and supportive supervision (Dousin et al., 2019). Besides, there is ample empirical evidence that job satisfaction can positively influence employee job performance (Krishnan et al., 2018; Zhao et al., 2019; Abdirahman et al., 2020). Based on the above research findings, the following hypotheses have been developed:

Hypothesis 2: Work-life balance has a positive effect on job satisfaction.

Hypothesis 3: Job satisfaction has a positive influence on job performance

Job satisfaction refers to the positive attitude felt by an employee toward the company where they work (Luthans et al., 2007; Tschopp et al., 2014). It combines cognitive and affective responses to the disparity between what an employee wants and what they get (Cranny et al., 1992). Previous research has often linked a person’s job satisfaction with their behavior at work (Crede et al., 2007). It is argued that employees would be more committed to their jobs if they found them satisfying and enjoyable (Noah and Steve, 2012). Employee job satisfaction is influenced by an organization’s commitment to work-life balance, and satisfied employees are more likely to invest their time and effort in the development of the organization (Dousin et al., 2019) in exchange for the support they received (Krishnan et al., 2018; Abdirahman et al., 2020). Previous research found that employee work-life balance increases employee job performance by positively influencing psychological well-being (Haider et al., 2017). Dousin et al. (2019) found that job satisfaction mediates the relationship between employee work-life balance and job performance in a medical context. Since work-life balance has been seen as an influencer of job satisfaction (Victoria et al., 2019) and job satisfaction influences employee job performance (Dormann and Zapf, 2001; Saari and Judge, 2004; Crede et al., 2007; Luthans et al., 2007; Tschopp et al., 2014; Krishnan et al., 2018; Zhao et al., 2019; Abdirahman et al., 2020). Thus, based on the above research findings, this study offers the following hypothesis:

H4: Job satisfaction significantly mediates the relationship between work-life balance and job performance.

### Family Supportive Supervisor Behaviors

Hammer et al. (2009) define family-supportive supervisor behaviors (FSSB) as the emotional, instrumental, role-modeling, and creative work-family management supportive behaviors that the supervisors provide to ensure employee effectiveness and satisfaction on and off the job. It refers to an employee’s perception of their supervisor’s positive attitude toward them (Clark et al., 2017). Supervisory support could be formal or informal (Achour et al., 2020). It is critical in developing flexible work arrangements (Suriana et al., 2021).

Supervisory supportive behavior is very important for ensuring work-life balance and achieving organizational goals. It has been shown to reduce work-family spillover (García-Cabrera et al., 2018) by increasing employee job satisfaction autonomy and reducing work pressure (Marescaux et al., 2020). The flexibility and independence generated by FSSB help to reduce work-family conflict (Greenhaus et al., 2012) by increasing employees’ control over their work (Marescaux et al., 2020) and allowing them to strike a balance between their work and family life (Heras et al., 2021). Employees who believe their managers care about their personal and professional lives are more likely to improve their performance and meet supervisory objectives (Rofcanin et al., 2018). In a university-based study, Achour et al. (2020) showed how supervisory support positively moderates the relationship between a female academic’s work-family demands and perceived well-being. Kim et al. (2017) show that supervisory support can strengthen the relationship between deep acting and job performance, exacerbating the negative relationship between surface acting and job performance. Therefore, this study argues that, in an organization, when work-life balance is valued, supervisory support might influence employees’ positive perception, and the effect of work-life balance strategies and job satisfaction on job performance will be greater.

Hypothesis 5: Family-supportive supervisor behaviors will strengthen the positive effect of work-life balance on job performance.

Hypothesis 6: Family-supportive supervisor behaviors will strengthen the positive effect of job satisfaction on job performance.

## Methods and Results

The current study has adopted a quantitative approach to determine the causal relationship of a phenomenon or problem-solving understudy to see how far the influence of exogenous variables extends to endogenous variables. The current study has also developed and distributed structured questionnaires to around 600 employees who work in SMEs in Indonesia.

To obtain and collect data, the study employed a non-probability method, namely purposive sampling. Purposive sampling is limited to certain types of people who can provide the desired information, maybe because they are the only ones who have it, or perhaps they fit the criteria set by the researcher (Sekaran and Bougie, 2017). The selected sample is employees who work in SMEs that already have an employee recruitment system, have supervisors, and are married. The sample size was taken as many as 400 samples with consideration of the adequacy of the sample statistically to get a power of 0.8 with an alpha of 0.05. The sample was repeated at least five times until 20 items were observed (Hair et al., 2015). The demographic profile of the respondents is presented in Table 1. The majority of the respondents were male (57%), aged 26–35 (50.5%), had one child (30%), were senior high school graduates (42.5%), and had 2 to 10 years of experience (43.2%). Furthermore, measurements and variables are presented in Table 2. The construct measurement items are reflective in nature.

**TABLE 1:** Characteristics of respondents.

Respondents	Frequency	Percent (%)
**Gender**		
Male	228	57%
Female	172	43%
**Age (years)**		
16–25	116	29%
26–35	202	50.5%
36–50	62	15.5%
51–70	20	5%
**Children**		
9	1	0.2%
7	1	0.2%
6	4	1.0%
5	7	1.8%
4	22	5.5%
3	27	6.8%
2	103	25.8%
1	120	30.0%
0	115	28.8%
**Education**		
Primary school	4	1%
Junior high school	10	2.5%
Senior high school	170	42.5%
Diploma	90	22.5%
Bachelor degree	126	31.5%
**Tenure (years)**		
<1	72	18%
1–2	101	25.2%
2–10	173	43.2%
>10	54	13.5%

**TABLE 2:** Summary for convergent validity and internal consistency reliability.

Constructs/Items	LF	CA	ρ_*A*_	CR	AVE
**Work-life-balance** (Talukder et al., 2018)		0.897	0.897	0.936	0.830
I have enough time for my family and friends	0.911				
I have enough time to carry out personal matters	0.904				
I have enough time to fulfill my personal interests	0.918				
**Family supportive supervisor behavior** (Bosch et al., 2018)		0.820	0.830	0.893	0.735
My supervisor makes me feel comfortable talking to him/her about my conflicts between work and non-work	0.812				
My supervisor demonstrates effective behaviors in how to juggle work and non-work issues	0.879				
My supervisor works effectively with employees to creatively solve conflicts between work and non-work	0.879				
**Job satisfaction** (Talukder et al., 2018)		0.907	0.913	0.925	0.607
My job is like a hobby to me	0.788				
My job is usually interesting enough to keep me from getting bored	0.820				
I feel that I am happier in my work than most other people	0.848				
I like my job better than the average worker does	0.842				
I find real enjoyment in my work	0.853				
**Job performance** (Talukder et al., 2018)		0.888	0.891	0.918	0.690
I meet formal performance requirements of the job	0.714				
I fulfill responsibilities specified in the job description	0.758				
I engage in activities that can positively affect my performance evaluation	0.709				
I perform tasks that are expected of me	0.814				
I can make constructive suggestions to the overall functioning of my work group	0.831				
I encourage others to try new and more effective ways of doing their jobs	0.805				

*LF = Loading’s factor; CA = Cronbach’s Alpha; ρ_A_ = rho_A; CR = Composite Reliability; and, AVE = Average Variance Extracted.*

### Empirical Estimations and Results

We employ the Partial Least Square (PLS) method to test hypotheses, considering variables’ direct, indirect, and total effects. PLS was chosen because the method of solving structural equation modeling (SEM) with PLS, which in this case fits the research objectives, is more appropriate than other SEM techniques. PLS is an analytical method that is not based on many assumptions (Hair et al., 2015). Finally, we employ PLS-SEM because of its applicability and effectiveness in both exploratory and confirmatory research and prediction (Chin and Dibbern, 2010; Ringle et al., 2012). To cope with missing values, we consider the mean replacement strategy (Wesarat et al., 2018). The parameters of the measurement and structural models are computed in accordance with the recommendations of Hair et al. (2014). Hypothesis testing is done by looking at the *p*-value generated by the inner model. This test is carried out by operating bootstrapping on the SmartPLS 3.0 program to obtain the relationship between exogenous and endogenous variables.

#### Measurement Model Evaluation

The measurement model has been evaluated in this study based on internal consistency, construct validity, and instrument reliability. The composite reliability can be used to assess the reliability of a variable’s indicators. With its indicators, there is a latent loading factor value. The loading factor is the path coefficient that connects the latent variable to the indicator. If an indicator has a composite reliability value greater than 0.6, it can fulfill reliability requirements. Cronbach’s alpha needs to be taken into account in the reliability test using the composite reliability approach. If a value has a Cronbach’s alpha value better than 0.7, it is deemed to be consistent (Hair et al., 2014). Convergent validity testing reveals the average variance extracted value (AVE), which should be greater than 0.6 Hair et al. (2014). The discriminant validity test is carried out by examining the value of the cross-loading factor and the criterion of the heterotrait-monotrait correlation ratio (HTMT). The HTMT ratio should not exceed 0.85 (Henseler et al., 2015). Finally, the multi-collinearity test focuses on determining if there is a relationship between exogenous variables. The tolerance and variance inflation factor (VIF) values are used to analyze the extent of collinearity. A VIF value of less than 10 indicates the presence of a collinearity-free indicator. Multi-collinearity is not an issue in our study as we used reflective measuring items.

**FIGURE 2:**
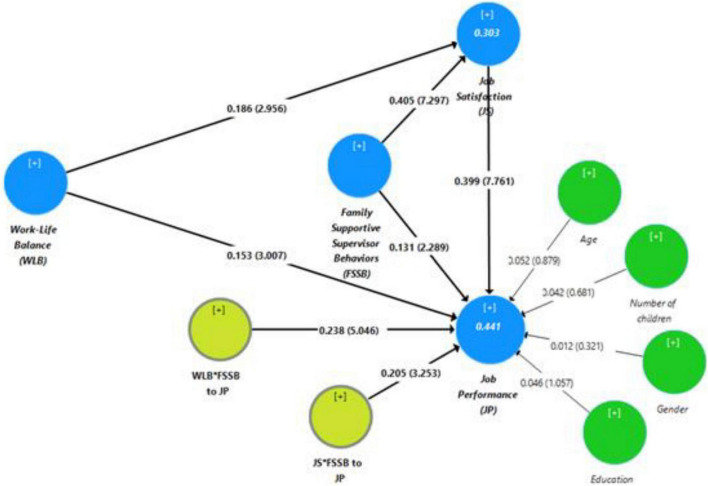
Result of structural model.

The results of convergent validity and composite reliability are presented in Table 2. We have observed that Cronbach’s alpha values for the construct lie between 0.820 and 0.907, which are above the cut-off value of 0.6, and all latent variables had Cronbach’s alpha values above 0.7. So, it can be concluded that the construct of our study has met the reliability criteria. Additionally, the indicator loadings range between 0.709 and 0.918, which has been presented in Figure 2, suggesting good content validity. Furthermore, the AVE value of our study variable is more than 0.50, indicating that convergent validity has been established. Furthermore, the results of discriminant validity are presented in Table 3. From the Fornell-Lacker Criterion in Panel A of Table 3, we noted the square roots of the AVE values (bold) are higher than the latent construct correlation. We also found that the HTMT ratio in Panel B of Table 3 between variables was less than 0.85. Henceforth, the Fornell-Lacker Criterion and HTMT ratio indicates the discriminant validity of the construct. In panel C of Table 3, the correlation between constructs is less than 0.90, showing no multicollinearity issue in the model (Pallant, 2011; Hair et al., 2013).

**TABLE 3:** Discriminant validity and latent variable correlation.

Constructs	FSSB	JP	JS	WLB
**Panel A: Fornell-lacker criterion**				
Family Supportive Supervisor Behaviors (FSSB)	**0.857**			
Job Performance (JP)	0.431	**0.779**		
Job Satisfaction (JS)	0.521	0.573	**0.831**	
Work-Life Balance (WLB)	0.545	0.388	0.421	**0.911**
**Panel B: Heterotrait-monotrait ratio (HTMT)**				
Family Supportive Supervisor Behaviors (FSSB)	1.000			
Job Performance (JP)	0.493	1.000		
Job Satisfaction (JS)	0.605	0.629	1.000	
Work-Life Balance (WLB)	0.637	0.425	0.471	1.000
**Panel C: Latent variable correlation**				
Family Supportive Supervisor Behaviors (FSSB)	1.000			
Job Performance (JP)	0.431	1.000		
Job Satisfaction (JS)	0.521	0.573	1.000	
Work-Life Balance (WLB)	0.545	0.388	0.421	1.000

*The square roots of the AVE values (bold) are higher the latent construct correlation.*

#### Structural Model Evaluation

Once the measurement model had met all the thresholds, the next step was to test the structural model. The r-square (reliability indicator) for endogenous components can be used to evaluate the structural model. The goal of variance analysis (R2) is to identify how exogenous variables affect endogenous variables. Figure 2 shows that R^2^ of 0.44 of job performance indicates that work-life balance, family-supportive supervisor behaviors, and job satisfaction explain 44 percent of the job performance variable, while the remaining 56 percent is explained by outside factors. Job satisfaction’s R^2^ of 0.304 indicates that work-life balance, family-supportive supervisor behaviors, and job performance explain 30.4 percent of the job satisfaction variable. In contrast, the remaining 69.6 percent is explained by components other than those explored in this study. The R^2^ of the endogenous variables job performance and job satisfaction in our study model is greater than 20%, indicating a good model (Hair et al., 2014).

#### Hypothesis Testing

For Hypothesis testing, resampling with bootstrapping can be used to compute the statistical *t* value. This study considered 5,000 sub-sample for bootstrapping and a two-tail significance level with biased correction. The empirical results for hypothesis testing are presented in Figure 2 and Table 4. Our hypotheses are supported by the empirical results at the significance level of 5%.

**TABLE 4:** Results for direct effects, mediating effect, and moderating effect.

Relationships	Std-Beta	STDEV	T Statistics	*P*-Values	95% BCa Confidence Interval*		Decisions
					Lower	Upper	
**Control variables**							
Age→ Job Performance (JP)	0.057	0.059	0.879	0.380	–0.074	0.157	NA
Education→ Job Performance (JP)	0.044	0.043	1.057	0.291	–0.037	0.133	NA
Gender→ Job Performance (JP)	0.012	0.039	0.321	0.748	–0.063	0.087	NA
Number of children→ Job Performance (JP)	0.037	0.062	0.681	0.496	–0.071	0.167	NA
**Direct effects**							
H1: Work-Life Balance (WLB)→ Job Performance (JP)	0.152	0.051	3.007	0.003	0.056	0.256	Supported
H2: Work-Life Balance (WLB)→ Job Satisfaction (JS)	0.187	0.063	2.956	0.003	0.062	0.307	Supported
H3: Job Satisfaction (JS)→ Job Performance (JP)	0.401	0.051	7.761	0.000	0.294	0.492	Supported
Family Supportive Supervisor Behaviors (FSSB)→ Job Performance (JP)	0.133	0.057	2.289	0.022	0.012	0.238	Supported
Family Supportive Supervisor Behaviors (FSSB)→ Job Satisfaction (JS)	0.405	0.055	7.297	0.000	0.294	0.511	Supported
**Mediating effect**							
H4: Work-Life Balance (WLB)→ Job Satisfaction (JS)→ Job Performance (JP)	0.075	0.028	2.639	0.008	0.024	0.134	Supported
**Moderating effects**							
H5: WLB × FSSB to JP→ Job Performance (JP)	0.235	0.047	5.046	0.000	0.141	0.324	Supported
H6: JS × FSSB to JP→ Job Performance (JP)	0.206	0.063	3.253	0.001	0.080	0.323	Supported

*Significant at the level of 0.05.*

## Conclusion and Discussion

### Theoretical Implications

Employees who have a poor work-life balance suffer from reduced productivity and low employee performance (Naithani, 2010). In contrast, employees with a healthy work-life balance have improved job performance (Roberts, 2008; Ryan and Kossek, 2008). In this regard, our findings demonstrate that the direct effect of work-life balance on job performance is significant with a coefficient of 0.152 (T-statistic of 3.007), suggesting a positive relationship between work-life balance and job performance. These empirical results also suggest that the employee’s job performance will also increase with a higher work-life balance. The respondents in the study also commented on their readiness to be flexible at work when needed, and they underlined that they are not ready to sacrifice their personal lives for work. Thus, the empirical findings lend strong support to our hypothesis H1. Our results are in line with the social exchange theory that a balanced proportion of time given by an employee to work-life and life-outside of work will make the employee more productive (Brough et al., 2008; Roberts, 2008; Ryan and Kossek, 2008; Hofmann and Stokburger-Sauer, 2017). In support of the WLB and performance nexus, French et al. (2020) and Haar et al. (2014) stated that a high work-life balance also makes individuals yield to their higher job performance. Therefore, SMEs need to create a work-life balance supportive culture in the organization in order to bring out employees’ best performances, which could lead to better firm performance. The fact is that the entanglements between work and family are a significant source of psychological discomfort for employees (Cegarra-Leiva et al., 2012), which causes poor performance. Additionally, Lamane-Harim et al. (2021) suggested that WLB could lead to better employee outcomes in Spanish SMEs. As a result, both employees and employers must work together to foster a work-life balance-supportive culture in the organization, which is especially difficult in the SME sector.

According to Victoria et al. (2019), satisfied and prosperous family life could lead to success and satisfaction at work. Therefore, the importance of work-life in employee job satisfaction is indicated in the literature (Dousin et al., 2019). Concerning that affirmation, this study’s evidence demonstrates that the effect of work-life balance on job satisfaction is significant with a coefficient of 0.187 (with a T-statistic value of 2.95), which is indicative of a positive relationship between work-life balance and job satisfaction. This finding implies that with a higher work-life balance, the job satisfaction of employees will also increase. Henceforth, the current results are strongly supported by hypothesis H2. These findings are in line with Haar et al. (2014); Dousin et al. (2019), and many others. Their studies also found that work-life balance has a positive effect on job satisfaction; namely, the higher the work-life balance, the higher the job satisfaction of employees. Flexible working hours, given autonomy, and company policies that support the creation of a balance between work and personal life will lead to higher job satisfaction (French et al., 2020). Feeney and Stritch (2019) stated that family-friendly policies and a culture of family support are essential in generating a healthy work climate. Henceforth, job satisfaction will increase. Additionally, creating a family-supporting culture, flexible working hours, and autonomy could not be done in the SME industry as the working environment is different from that of large organizations. However, suppose SMEs take the initiative to create some sort of flexible working hours and give some autonomy depending on their position inside the company. In that case, the employees could be more satisfied, especially if the primary intention is to increase employee productivity and performance. In support of this statement, our findings have found a positive influence of job satisfaction on job performance.

Job satisfaction and job performance are widely studied relationships in HRM and organizational contexts. Most studies have discovered a positive relationship between job satisfaction and job performance (Dormann and Zapf, 2001; Saari and Judge, 2004; Crede et al., 2007; Luthans et al., 2007; Tschopp et al., 2014; Krishnan et al., 2018; Jermsittiparsert et al., 2019; Zhao et al., 2019; Abdirahman et al., 2020). As expected, in the current context of the study, we also found that the effect of job satisfaction on job performance is significant, with a coefficient of 0.401 (with a T-statistic value of 7.761). Hence, the current empirical findings lend strong support to H3 that job satisfaction will increase job performance. Therefore, in line with the extant studies, we also argue that SMEs should attempt to keep employees satisfied with their jobs so they can generate their best performance. The organizational theory suggests that perceived job satisfaction makes employees more committed toward their jobs, hence better output. In the SME case, work–life balance and a supportive culture could play an important role in making employees more committed and satisfied, which will increase job performance. Our hypothesis rectifies this assertation that H3 work-life balance has positive effects on job satisfaction.

In their study, Haider et al. (2017) have discussed how work-life balance increases employee job performance *via* influencing psychological well-being. Job satisfaction is one of the main components of psychological well-being at the workplace. Therefore, on the mediating role of job satisfaction, our findings demonstrate that the relationship between work-life balance and job performance is mediated by job satisfaction (with a coefficient of 0.075 and a T-statistic value of 2.64). Since there is a direct relationship between work-life balance and job performance, it can be concluded that the mediation is a partial mediation rather than a full one. Thus, our hypothesis H4 is accepted. The current empirical findings also support the past empirical studies, as Dousin et al. (2019) found the mediation role of job satisfaction between employee work-life balance and job performance in a medical context. Hence, our findings imply that work-life balance improves job performance by increasing job satisfaction.

Family supportive supervisor behaviors (FSSB) in the organization are about work-family spillover (García-Cabrera et al., 2018) by boosting employee job satisfaction autonomy and minimizing work pressure (Marescaux et al., 2020). Hence, it has been able to increase job satisfaction and performance. In this regard, although we do not hypothesize the direct effect of family-supportive supervisor behaviors, our findings confirm that FSSB positively influences job satisfaction and performance. Therefore, the existence of FSSB is essential to improve employees’ job satisfaction and job performance. Hence, these findings agree with the past studies that present a positive influence of FSSB on job satisfaction and job performance (Rofcanin et al., 2018; Talukder et al., 2018; Campo et al., 2021). Henceforth, these findings confirm the assertion of social exchange theory and organizational support theory that supervisors’ formal and informal support further increase employees’ attitude toward the job, which improves job satisfaction and job performance (Talukder et al., 2018).

Furthermore, our empirical results indicate that the interaction between FSSB and work-life balance positively affects job performance (with a coefficient of 0.235 and a t-statistic of 5.04). These findings suggest that when FSSB interacts with work-life balance, it attenuates the link between work-life balance and job satisfaction and job performance. As a result, the current findings provide significant support for our hypothesis H5. Kim et al. (2017) discovered that supervisory support could increase the link between deep acting and work performance. On the other hand, Alias (2021) suggest that supervisory support cannot moderate the relationship between flexible work arrangements and employee performance. Our findings, however, offer evidence that contradicts the assertion of Alias (2021), in which we demonstrated that there could be moderating effects on the relationship between work-life balance and job performance. Hence, our finding adds novel evidence in the area of work-life balance and job performance. Again, these findings reinforce the need for a work–life balance supportive culture in the organization, as it could facilitate supervisory actions to a certain degree in supporting employees’ family and personal life.

Based on hypothesis H5, we concurred on the moderating impact of FSSB on the link between job satisfaction and job performance. We evaluated the moderating influence of FSSB on this relationship. The current study’s empirical findings indicate that the interaction effects of FSSB and work satisfaction on job performance are relatively positive (with a coefficient of 0.206 and a t-statistic of 3.25). These findings suggest that when FSSB interacts with work-life balance and job satisfaction, it moderates the link between work-life balance and job satisfaction and job performance. Hence, the current empirical results verify our claim and offer substantial support for Hypothesis H6. The interaction effects are reasonably sensible in that when employees are satisfied and believe that they will receive the required support from their boss while coping with family or personal concerns. As a result, when the level of belief and job satisfaction rises, so does the level of job commitment and engagement, resulting in higher job performance. In this regard, the current study contributes to the body of evidence on the FSSB’s moderating effects on job satisfaction and performance.

### Practical Implications

In support of the WLB-performance nexus, several studies have indicated that an excellent work balance also leads to more extraordinary job performance. Thus, SMEs must foster a work–life balance-friendly culture to bring out the best in their employees, which may contribute to improved business/firm performance. In reality, the entanglements between work and family are a major source of psychological distress for employees, resulting in poor performance. Henceforth, the implementation of various WLB practices is suggested for Indonesian SMEs, particularly those not required by regulation or legal minimum to fulfill the needs of all employees. Furthermore, we also recommend that firms should provide separate WLB practice alternatives for men and women because the impacts of WLB on job satisfaction are varied, as suggested by Lamane-Harim et al. (2021). Furthermore, family-supportive supervisor behaviors are important for promoting employees’ performance. Therefore, firms and supervisors provide some support to employees to handle and overcome family-related issues. In this regard, our findings emphasized the need to establish a work–life balance supportive culture in the firm as it might assist supervisory activities in supporting workers’ family and personal life to a different extent. In addition, managers may gain useful knowledge to create efficient job systems to improve job performance in SMEs, taking into account the relevance of work-life balance, family supportive supervisor behaviors, and job satisfaction. Individuals in SMEs can increase job performance by balancing their work and personal life. The impact of SMEs on employee work-life balance and performance is a fascinating topic. As a result, work-life balance will have a bigger impact on the organization’s overall performance.

### Limitation and Future Research

We propose that this research be expanded into a longitudinal study in the future, providing a greater grasp of the issue. However, the findings may not be generalizable, and the results must be interpreted in light of the evolving context and economic conditions in which the study was done. Additionally, future studies should look into religiosity as a moderator of the relationship between WLB and job satisfaction and performance. It’s important to think about becoming a moderator since employees who have a strong understanding of religion and put it into practice have a good sense of self-control. It could have a different effect when attempting to explain the link between work-life balance and job performance. Stress and anxiety are one of the most essential factors to consider when attempting to explain the link between WLB and job performance. Many employees may feel stressed and anxious about their professional and personal development while working in SMEs. As a result, as moderators in this association, it may be an important aspect to investigate in future research. Finally, future research should look at deviant behavior as a result of work-life balance and job satisfaction. Employees with a poor work-life balance and dissatisfaction are more likely to engage in deviant behavior.

## Data Availability Statement

Data will be provided by the first author upon request.

## Author Contributions

All authors listed have made a substantial, direct, and intellectual contribution to the work, and approved it for publication.

## Conflict of Interest

The authors declare that the research was conducted in the absence of any commercial or financial relationships that could be construed as a potential conflict of interest.

## Publisher’s Note

All claims expressed in this article are solely those of the authors and do not necessarily represent those of their affiliated organizations, or those of the publisher, the editors and the reviewers. Any product that may be evaluated in this article, or claim that may be made by its manufacturer, is not guaranteed or endorsed by the publisher.
